# Mr. Bellot's Case of Suppression of Urine

**Published:** 1808-03

**Authors:** Abraham Bellot

**Affiliations:** Oldham, Lancaster


### *To the Editors of the Medical and Physical Journal.*

GENTLEMEN,

ON November 29, 1804, I was requested to visit Edward Walker, who was labouring under a total suppression of urine; from which he was suffering the most excruciating pain imaginable. Upon inquiry, I found he had had a fistula in perineo for several months, through which the greatest part of his urine flowed. Notwithstanding he was in this unpleasant situation, he continued his usual employment; and, regardless of the consequence, was frequently intoxicated. At length, the urine insinuated itself into the cellular membrane, and the scrotum was completely distended with urine. This produced a violent inflammation, which extended from the perinæum as far as the navel, covering the whole of the lower part of the abdomen. No urine could now pass either through the small openings in the perineum, or along the urethra. In this deplorable situation I found the patient; who was uttering the most pitiable groans, and praying that he might speedily be released from his sufferings. The introduction of either catheter or bougie was utterly impracticable; and, perceiving the man could not exist long in this situation, I immediately proposed puncturing the scrotum, to which he would not consent, saying he would rather die. I used all entreaties, but in vain, and was obliged to content myself with ordering him an anodyne mixture and lotion. I visited him again late in the evening, but still was unsuccessful in my attempt to prevail upon him to submit to the operation. Upon examining the scrotum, I found it blistered, and of a livid appearance; which shewed that mortification was rapidly advancing. I opened a large blister and let out some water, and left him in the most extreme torture, expecting he would scarcely survive the night; but visiting him the next morning, I found him much relieved; the scrotum had burst, and a quantity of water rushed from him immediately; through this opening the urine continued to flow. The mortification now appeared to be very extensive outwardly, and I judged it had also taken place in the bladder; but fortunately this was not the case; the greatest part of the skin of the scrotum, with the septum scroti, sloughed away, which laid the testicles quite bare, and brought into view the opening in the urethra, through which the urine came, and was always preceded by the discharge of a large quantity of matter. I introduced a bougie into the end of the urethra, but could not pass it farther down than to this opening, which was about 2½ inches from the end of the urethra; the bougie I distinctly felt through the opening in the urethra.

As the bougie could not be passed farther down, and the urine flowing freely through this opening over the testicles, I concluded the urine had found a passage along the cellular membrane; but all my endeavours to discover this passage, by bougie or probe, proved fruitless. Though I could not satisfy myself on this point, I determined, by the daily introduction of a small bougie from the end of the urethra, to overcome the obstruction, and bring about the natural passage for the urine if possible. On the lower part of the abdomen, there was a large collection of matter, and a sloughing of the cellular membrane took place:

place: but by the introduction of a seton this extensive sinus was healed. It ought to be observed, that, from the time of the bursting of the scrotum, the patient took opium, bark, and cordials, and the stale-beer-ground poultice was applied over the mortified parts; and when the sloughing took place, common dressings. By persevering in the introduction of bougies, the stricture, which was immediately below the opening in the urethra was overcome. But this was not the only difficulty to be surmounted; for, on passing the bougie along the urethra, another stricture was met with in the perineum, where the fistulous opening was, but which was now closed. As the urine continued to flow freely over the wound, which wore a very healthy appearance, and the patient's health daily improving, I had great hopes of overcoming this second stricture; bougies were daily introduced, till a small one was made to pass into the bladder. By gradually increasing the size of the bougie, the urethra was kept sufficiently dilated; and before the wound healed, the urine flowed partly through the end of the penis, and partly by the aforesaid opening. Things continued in this state for some time; at length, however, the wound healed up, and the urine flowed regularly by the proper passage, though in a small stream; and if the introduction of the bougie was omitted, even one day, the difficulty of voiding the urine became increased. The introduction of bougies was regularly persevered in till the latter end of March 1805, when they were discontinued, in consequence of my being called to Lancaster; and to my great astonishment, on my return, which was in about a week, I found my patient married.

I am, &c.

ABRAHAM BELLOT.

*Oldham, Lancaster, Feb.* 12, 1808.

